# Application of convolutional neural networks for prediction of disinfection by-products

**DOI:** 10.1038/s41598-021-03881-w

**Published:** 2022-01-12

**Authors:** Nicolás M. Peleato

**Affiliations:** School of Engineering, University of British Columbia Okanagan, 1137 Alumni Ave., Kelowna, BC V1V 1V7 Canada

**Keywords:** Environmental sciences, Chemistry, Engineering

## Abstract

Fluorescence spectroscopy can provide high-level chemical characterization and quantification that is suitable for use in online process monitoring and control. However, the high-dimensionality of excitation–emission matrices and superposition of underlying signals is a major challenge to implementation. Herein the use of Convolutional Neural Networks (CNNs) is investigated to interpret fluorescence spectra and predict the formation of disinfection by-products during drinking water treatment. Using deep CNNs, mean absolute prediction error on a test set of data for total trihalomethanes, total haloacetic acids, and the major individual species were all < 6 µg/L and represent a significant difference improved by 39–62% compared to multi-layer perceptron type networks. Heat maps that identify spectral areas of importance for prediction showed unique humic-like and protein-like regions for individual disinfection by-product species that can be used to validate models and provide insight into precursor characteristics. The use of fluorescence spectroscopy coupled with deep CNNs shows promise to be used for rapid estimation of DBP formation potentials without the need for extensive data pre-processing or dimensionality reduction. Knowledge of DBP formation potentials in near real-time can enable tighter treatment controls and management efforts to minimize the exposure of the public to DBPs.

## Introduction

The use of fluorescence spectra for improved water quality monitoring^1^ and as a process analytical technology for bioprocesses, food, and pharmaceutical production, has become increasingly popular^2,3^. Fluorescence signatures are highly dependent on molecular structure, size, and environmental conditions, and therefore can be used to provide insight into chemical composition and properties^4^. The sensitivity and specificity of fluorescence analysis, coupled with the potential real-time monitoring capabilities, fluorescence has applicability to a wide variety of process control applications^5^.

One promising application is the improved prediction and control of disinfection by-product (DBP) formation from drinking water treatment with chlorine. Chlorination is the most common disinfectant used worldwide. However, when chlorine reacts with natural organic matter (NOM), present in all natural water sources, various by-products of health concern are formed^6^. Although many unique DBP species can be formed with varying public health risk, only specific groupings are commonly monitored and regulated in drinking water, including trihalomethanes (THMs) and haloacetic acids (HAAs). The monitoring frequency of regulated DBPs is generally low, with sampling only required once every 3 months for water systems in the United States and Canada^7^. Low frequency sampling is due to the current reliance on external laboratories to carry out DBP analysis implying significant cost and time delays^8^.

The cost and time-delays involved in DBP analysis severely limit the ability of utilities to control the water treatment process for minimizing DBP formation, and has spurred efforts to develop models that can predict DBP formation potential on a more frequent basis^9–13^. Since DBPs are formed from the reaction of chlorine and NOM, models must incorporate a measure of NOM. However, NOM is a chemically diverse grouping of organic molecules whose characteristics are dependent on the surrounding environment. As such, the breadth of potential NOM characteristics and the spatial and temporal variability results in significant challenges in identifying an optimal measure that can capture this complexity and reactivity with chlorine^14^. Fluorescence spectroscopy has considerable potential for the prediction and monitoring of DBP precursor material. Many NOM compounds fluoresce and fluorescence measures can capture some chemical characteristics of NOM^15^. Previously, fluorescence has been used with success to predict or identify correlations with regulated DBPs^16–18^, as well as unregulated or by-products of emerging concern such as chloral hydrate^19^ and haloacetonitriles^20,21^.

A common challenge to implementing fluorescence as a monitoring tool is the high-dimensionality and superposition of the resulting emissions. When utilizing fluorescence spectra collected at iterated excitation/emission wavelengths, a dimensionality reduction approach is often used to simplify excitation–emission matrices (EEMs)^15^. By identifying a few underlying components that explain most of the variance in the data, the hypothesis is that noise is reduced, and subsequent modelling using a reduced dimensionality improves prediction. A basic simplification or dimensionality reduction approach would be to select peaks or regions in the fluorescence spectra where regional integration or peak fluorescence can be determined. While this type of expert guided approach has been used extensively in the past, discarding the majority of collected data neglects the richness of information contained. For complex systems such as those that include identifying natural organic matter (NOM) in water, organic fluorophores with similar chemical structures are not easily distinguished in the spectra. The use of principal component analysis (PCA) or parallel factors analysis (PARAFAC) has revealed underlying signals resembling fluorophores, which can be tied to spectral regions from which chemical properties can be inferred^15,22,23^. These analysis approaches are often limited to linear dimensionality reduction, so non-linear features such as Rayleigh or Raman scattering need to be removed from the spectra^24^. Furthermore, potential impacts of environmental conditions such as pH or temperature^4^, or possible charge-transfer interactions^25^ may invalidate the assumption of a linear relationship between fluorophore concentrations and fluorescence intensity. Inner filter effects are also prevalent, where incident excitation light and emitted fluorescence is quenched by other chromophores present in the sample, result in a non-linear intensity response^26^. Constraints imposed by the method can be helpful when derived from prior knowledge of the system, such as non-negativity of fluorescence emissions, reducing bias and possibly resulting in a more accurate depiction of underlying structures. However, these same constraints may limit the overall accuracy of reconstruction based on the condensed representation^27^.

It may be advantageous to directly use all data collected in fluorescence EEMs to limit potential errors introduced from dimensionality reduction. However, for water quality analysis, there have been limited studies that explore the use of full fluorescence EEMs without dimensionality reduction. Non-linear regression using high-dimensional inputs can be accomplished using neural networks. More recent work with Convolutional Neural Networks (CNNs or ConvNets) has shown this type of network structure is well suited to interpreting images or other tasks datasets with local groups of values that are highly correlated^28^. Instead of training weighted connections between every individual node, CNNs train spatial filters or kernels to identify small recurring features in the input space. The use of filters allows for parameter sharing, where trained weights are used throughout the input space and are not tied to specific input nodes, giving rise to spatial invariance of features^29^. Furthermore, CNNs typically employ pooling layers where outputs in specific locations are merged with nearby outputs, creating invariance to small distortions in the input and reducing the dimensionality of the representation^28,29^. CNNs have been successfully applied in chemometric applications such as interpreting Raman and mid-infrared spectra for identifying *Escherichia coli* and meats^30^, pharmaceuticals in tablets with near infrared spectra^31^, categorizing wines using infrared spectra^32^, and classification of manganese valence^33^. However, there has been no use of CNNs for interpreting 2D fluorescence spectra, and previous implementations have focused on 1D infrared or Raman spectra. Furthermore, the use of CNNs for fluorescence analysis of water quality has not been explored. It is hypothesized that the strengths of CNNs for processing and interpreting spatially dependent data will be well suited for 2D fluorescence spectra where local groups of values are highly correlated.

This paper investigates the use of deep NNs and CNNs to interpret fluorescence spectra for the prediction of DBP formation potential. The two major groups of regulated DBPs are assessed, THMs and HAAs including the individual species that made up these groups in the samples analysed (trichloromethane, bromodichloromethane, trichloroacetic acid, and dichloroacetic acid). A method to interpret the CNN results is also used to identify fluorescence regions that are most likely associated with high DBP formation potentials.

## Results

A dataset of DBP formation potentials and associated fluorescence EEM measurements were used to assess the capabilities of deep NNs and CNNs for water quality analysis. Water samples analyzed were from a pilot-scale treatment plant receiving river water. Samples were taken throughout a treatment train consisting of several unit processes including coagulation, flocculation, sedimentation, ozonation, advanced oxidation (peroxide and ozone), and filtration through anthracite or activated carbon. As such, the samples analyzed had a wide range of NOM concentrations and characteristics. Dissolved organic carbon varied from 2.6 to 6.3 mg L^−1^, and specific ultraviolet absorbance varied from 0.75 to 2.53 L mg^−1^ m^−1^ over all samples. DBP formation potentials were determined by maintaining a free chlorine residual of 1.5 mg/L for 24 h. Although all four chlorinated or brominated THM and nine HAA species could be detected, only trichloromethane (TCM), bromodichloromethane (BDCM), trichloroacetic acid (TCAA), and dichloroacetic acid (DCAA) were consistently identified at concentrations above detection limits.

### Multi-layer perceptron

**Figure 1:**
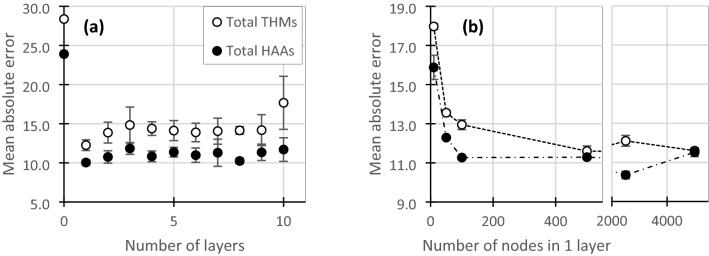
Mean absolute error (MAE) of prediction for a test set (n = 28) of THM concentrations and HAA concentrations using a MLP. (**a**) The effect of number of layers on performance, (**b**) the effect of number of nodes in 1 hidden layer on performance. Error bars represent 95% confidence intervals based on 8 random initializations of the network weights.

An iterative optimization approach was used to understand the impact of NN structure on overall performance. While many aspects of network structure can be optimized, the focus in this work was on the number of hidden layers (i.e. depth). A multi-layer perceptron (MLP) network was trained with an increasing number of layers to identify the degree to which network depth can improve prediction accuracy. Figure 1 shows the total THM and HAA predictions results given the number of layers in a MLP. The error bars in Fig. 1 represent the standard deviation of 8 repeated random initializations of the network. A network with 0 hidden layers is simply the input values (dimensions = 5632) connected to 1 output node. When the number of layers was increased, each layer's nodes were set to half of the previous layer. For example, with two hidden layers, hidden layer 1 would have 2816 nodes, and layer 2 would have 1408 nodes.

As observed in Fig. 1, MLPs with greater than 1 hidden layer did not improve network performance in predicting both total THMs and HAAs. Total THM mean absolute error was at a minimum with one hidden layer (MAE: 12.26 ± 0.34 µg/L; *p* < 0.02 compared to all other number of hidden layers) and total HAA error was also at a minimum with one hidden layer (MAE: 10.04 ± 0.33 µg/L, *p* < 0.03), although no significant difference between HAA performance with 1, 7, or 8 layers was found (*p* > 0.05). Similar results were observed for individual species (Table 1). Adding additional layers also came at the cost of increased variability between random network initializations. For example, the coefficient of variation (CV) increased from 3.3% for a one-layer MLP to predict total HAAs to 8.4% for 6 layers. Increased variability in performance could be due to the increased number of learnable parameters and the relatively small sample size used in this study. The structure chosen resulted in 15,870,977 trainable parameters with one hidden layer. Decreasing the number of nodes in 1 hidden layer, to effectively reduce the number of trainable parameters, demonstrated that reducing the number of nodes below 500 resulted in a decrease in performance and optimal performance for THMs was with 500 or 5000 nodes (*p* < 0.01 for all other comparisons) or 2500 for total HAAs (*p* < 0.01) (Fig. 1b). The lack of improvement of MLPs beyond one hidden layer is expected given the small data size and demonstrates that deep MLPs are unlikely to provide advantages in modelling small water quality datasets.

**Table 1:** Mean absolute error (MAE) of predictions on tests set for several model types. Range or error (±) is calculated as the 95% confidence interval, where applicable. Bolded numbers represent the optimal model for each species as determined by *t* tests at 95% confidence levels.

Disinfection by-product species	Range of DBP concentrations (μg L^−1^)	Mean absolute error (μg L^−1^)						
		MLP (1 layer)	CNN (1 layer)	CNN (4 pooling layers, 1 convolutional layer)	CNN (4 pooling layers, 4–5 convolutional layers)	PARAFAC-MLP	PCA-MLP	3-way PLS
Total THMs	26.5–208.2	12.3 ± 0.2	6.6 ± 0.1	6.1 ± 0.2	**5.6 ± 0.1**	18.7 ± 0.6	15.4 ± 0.5	15.9
Trichloromethane	24.0–174.3	8.8 ± 0.2	7.0 ± 0.6	4.9 ± 0.4	**3.4 ± 0.1**	16.6 ± 0.9	13.8 ± 0.5	12.6
Bromodichloromethane	13.6–62.6	6.3 ± 0.4	4.7 ± 0.2	4.1 ± 0.2	**3.9 ± 0.0**	6.5 ± 0.3	7.4 ± 1.2	6.4
Total HAAs	28.1–139.5	10.0 ± 0.2	4.5 ± 0.2	**4.2 ± 0.3**	**4.4 ± 0.1**	12.5 ± 0.2	12.4 ± 0.3	11.1
Dichloroacetic acid	17.7–85.8	7.8 ± 0.3	6.1 ± 0.7	4.8 ± 0.2	**4.2 ± 0.1**	6.1 ± 0.3	8.0 ± 0.3	8.9
Trichloroacetic acid	10.4–81.4	8.4 ± 0.2	5.9 ± 0.6	4.6 ± 0.1	**4.2 ± 0.1**	7.0 ± 0.3	6.8 ± 1.2	5.3

### Convolutional networks

**Figure 2:**
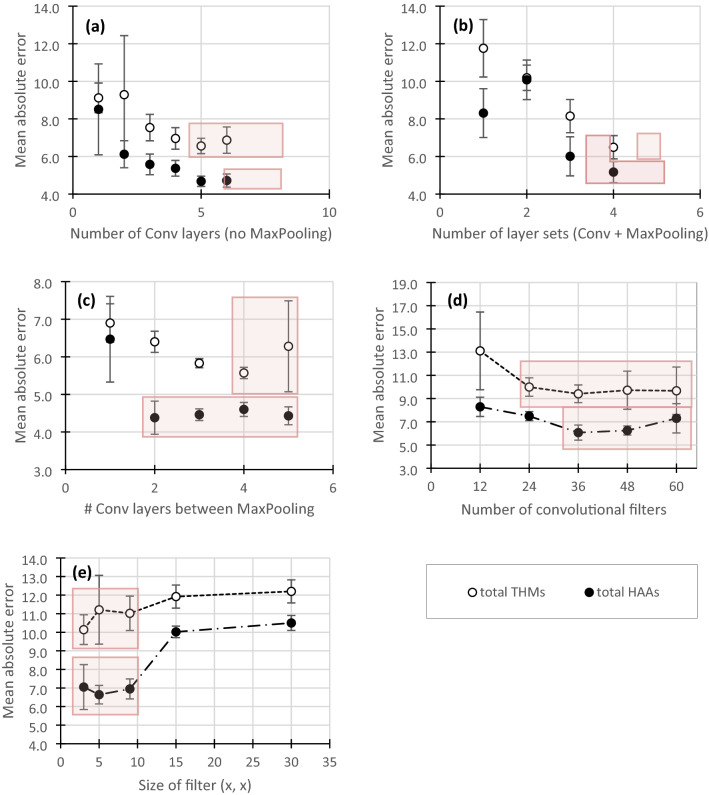
Impact of CNN structure and depth on MAE of test set predictions. (**a**) Varies the number of convolutional layers without any max pooling layers, (**b**) varies the number of layer sets with 1 convolutional layer followed by max pooling, (**c**) varies the number of convolutional layers between max pooling layers (4 max pooling layers in total), (**d**) varies the number of convolutional filters for 1 convolutional layer without max pooling, (**e**) varies the size of the receptive field for 1 convolutional layer without max pooling. Red boxes delineate areas of optimal performance, where all models within the box performed similarly based on *t* tests at 95% confidence levels.

In contrast to MLPs, prediction accuracy was minimized with increasing CNN network depth (Fig. 2). Network depth was investigated by increasing the number of convolutional layers and the number of layer sets (convolution followed by max pooling). Convolutional layers provide learned filters or kernels that identify small features in the spectrum, while max pooling layers decrease dimensionality and pool redundant features^28,29^. Including 4–6 hidden convolutional layers without pooling layers was found to optimize prediction accuracy compared to 0–3 layers (*p* < 0.03 for all comparisons) for THMs and 5–6 layers was optimal for HAAs (*p* < 0.01) (Fig. 2a). The performance of models with 4–6 layers were not found to be significantly different for THMs (*p* > 0.13) or 5–6 layers for HAAs (*p* > 0.80). Increasing the number of layer sets also improved performance compared for THMs (Fig. 2b). A further decrease in error of 15.1% for total THMs was significant when both the number of convolutional and max pooling layers were increased (*p* < 0.01), but the more marginal increase in HAA performance (5.3%) was not significant (*p* = 0.07) (Fig. 2c; Table 1).

It was also of interest to investigate the role of the size of receptive fields for each filter and the number of filters included in each convolutional layer. The receptive field size identifies the number of adjacent data points to be considered by each filter. Previous work in chemometrics has shown relatively large receptive fields to work well^32^ and could identify features that span over large areas of the spectra, however, expanding the filter size increases the number of trainable parameters. Alternatively, by including convolutional layers in sequence, the receptive field's effective size is expanded, minimizing the number of trainable parameters and including additional layers of non-linearity^34^. As such, the results suggest that larger receptive fields could improve CNN performance. However, increasing the receptive field size beyond (3, 3) for individual layers did not improve performance (*p* > 0.06 for sizes (3, 3), (5, 5), and (7, 7) for both THMs and HAAs) (Fig. 2e), and expanding receptive fields may be best accomplished by stacking convolutional layers in sequence. It is also of note that increasing the number of trained filters improved performance up to 24–36, after which no further changes were observed (*p* > 0.34) (Fig. 2d).

**Figure 3:**
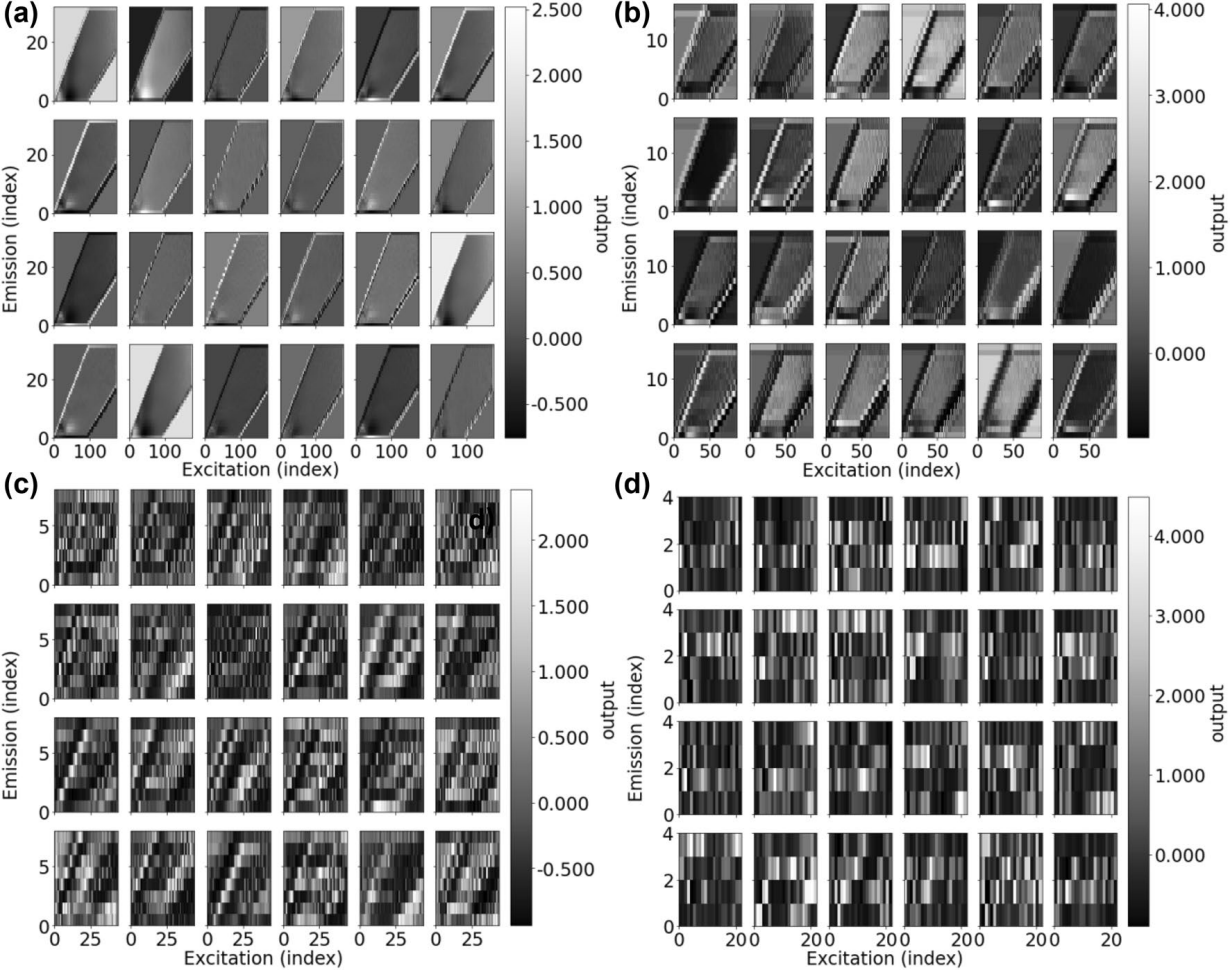
Feature maps of convolutional filters (24) from 4 convolutional layers chosen between max pooling layers. (**a**) First convolutional layer, (**b**) after the first max pooling, (**c**) after the second max pooling, (**d**) after the third max pooling. All max pooling was carried out over a (2, 2) window.

An example of the learned filters are shown in Fig. 3. CNNs create hierarchical representations of data showing how specific irrelevant spectral features are discarded and specific areas of the spectra needed to predict DBP concentrations are magnified^30^. The initial filter layer identifies large and smooth and broad features in the spectra. After pooling, feature maps become more coarse and more distinct patterns between filters can be discerned, highlighting specific areas of the spectra. In the last layer of feature maps, many filters highlight one constant emission level over several excitation bands (left to right).

The comparison of MLP and CNN structures shows a marked performance improvement using a convolutional approach (Fig. 2 and Table 1). Compared to the optimized MLP, a network with one single convolutional layer improved prediction accuracy by 54.6% for total THMs (*p* < 0.01; MAE reduced by 6.69 µg/L) and 55.9% for total HAAs (*p* < 0.01; MAE reduced by 5.61 µg/L) (Table 1). Improvements were also significant for individual species (39.1–61.5% reduction in MAE). In general, the most significant decrease in error was observed using CNNs, followed by adding several pooling layers. While adding multiple convolutional layers between pooling layers did further reduce error, the gains were more minor (MAE difference 0.25–1.48 µg/L) but statistically significant (*p* < 0.02). However, adding stacked convolutional layers resulted in a slight increase in error for total HAA prediction (− 0.23 µg/L). As such, the pooling of features and reduction of dimensions is likely key to best interpreting EEMs. It is hypothesized that the success of using pooling layers is due to building in dimensionality reduction and reducing the number of nodes in the final fully-connected layer.

### Model explanations

The objective of identifying model explanations was to confirm that that model predicts high or low concentrations of DBPs based on fluorescence features that are known or possibly associated with DBP precursors. There are scattering signals (i.e. not from organic material) or other potential artifacts from the sample analysis process that would bias the model to “know” concentrations of DBPs for incorrect reasons. The second objective was to identify fluorescence regions most highly associated with specific DBP formation potentials. This information could be used to further understanding of the characteristics of DBP precursors and potentially optimize treatment processes that preferentially remove compounds with those characteristics.

**Figure 4:**
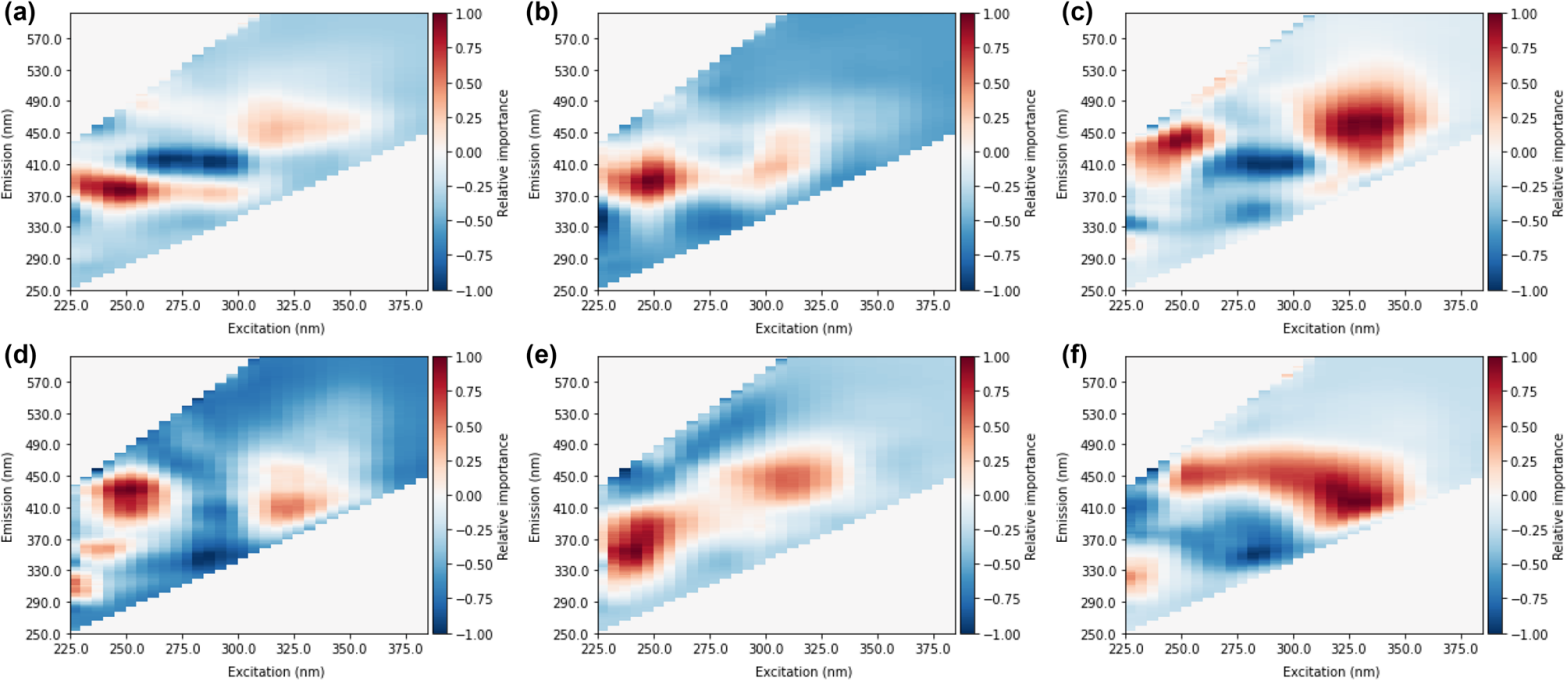
Heat maps from random occlusion of variable importance for CNN prediction of (**a**) total THMs, (**b**) trichloromethane, (**c**) bromodichloromethane, (**d**) total HAAs, (**e**) trichloroacetic acid, (**f**) dichloroacetic acid.

**Figure 5:**
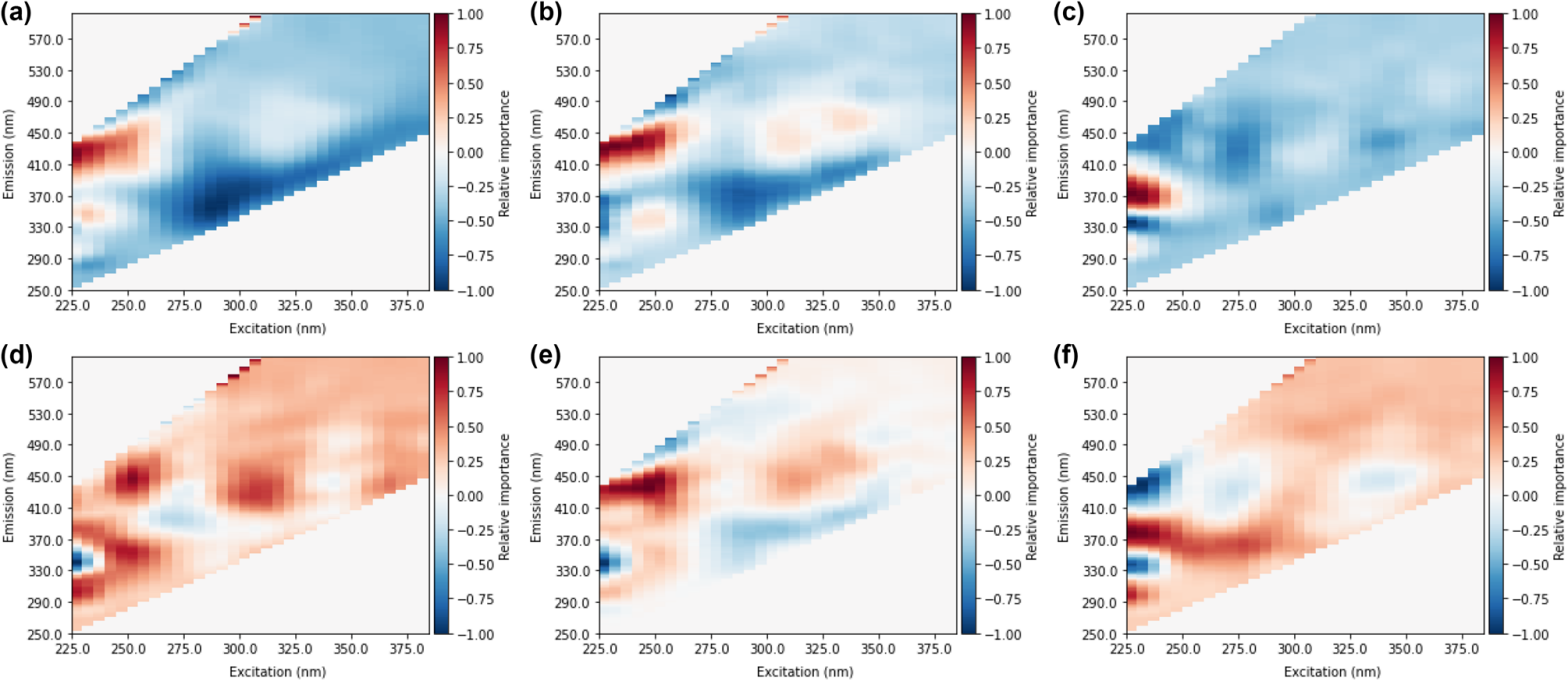
Heat maps from random occlusion of variable importance for MLP prediction of (**a**) total THMs, (**b**) trichloromethane, (**c**) bromodichloromethane, (**d**) total HAAs, (**e**) trichloroacetic acid, (**f**) dichloroacetic acid.

An occlusion method was applied to identify spectral areas that most significantly influence prediction accuracy. The occlusion method identifies spectral regions most relevant to a prediction by randomly occluding or setting a segment of all inputs in a specified region to 0. The error incurred due to this occlusion indicates how relatively important that specific area is to accurate predictions. The error was calculated as the difference between non-occluded and occluded predictions, and the direction or sign of the error was preserved. As such, positive values indicate that the model underestimated DBP formation with a specific patch occluded, and negative values indicate overestimated DBP formation. A total of 20,000 iterations of random patches per model were chosen to build the heat maps. A random approach to selecting the patch was taken to reduce any bias from neighbouring values since the variables included in each patch would change between iterations. Figure 4 shows the average heat maps identified from training deep CNNs on total DBPs and individual species. Likewise, Fig. 5 shows heat maps based on MLPs.

From occlusion heat maps of variable or spectra area importance (Fig. 4), it was observed that fluorescence in the area of approximately ex: 225–260 nm and em: 370–500 nm was most impactful of prediction accuracy for all DBPs (both total and individual species). A second common area of importance at ex > 300 nm and em > 400 nm was also observed. Fluorescence in these two regions is generally considered to be humic-like and fulvic-like material^35^. Several heat maps also show areas of importance in protein-like fluorescence regions (excitation: 230–250 nm, emission: 300–360 nm) associated with tryptophan-like or tyrosine-like fluorescence^35^. Spectral importance in these regions conforms well to expectations of DBP precursor type material that can fluoresce, generally thought to be aromatic humic-like or fulvic-like material^35^. The heatmaps provide evidence that the NNs are utilizing signals from regions that are reasonable for DBP prediction. Previous DBP prediction methods based on fluorescence data have utilized the same spectral regions^16,17,36^.

Compared to MLPs, CNN heat maps show broader areas of importance with more gradual changes (Figs. 4 and 5). Gradual changes conform with the expectation of fluorescence signals from fluorophores, and sharp changes are not typically associated with fluorescence from natural organic matter^15^. Furthermore, CNN heat maps emphasize higher excitation bands. Particularly for prediction of trihalomethanes, peaks at excitation > 300 nm were important for positive predictions, while MLP heat maps placed less importance on these areas. Several CNN heat maps show signals that have several excitation peaks, with limited changes in emission. For example, the BDCM heat map shows peaks at approximately 250 nm and 330 nm, with emissions constant at 450 nm. Multiple excitation peaks at one singular emission conform well with expectations of fluorescence from individual fluorophores, where multiple wavelengths can cause excitation, however, the emission is always from the lowest singlet state and therefore only at one wavelength^37^. The identification of areas of importance at several emission bands suggests several distinct fluorophores contributing to DBP formation potential rather than individual components.

It can also be observed that there is greater continuity of areas of importance with individual DBP species and the total levels. Since the individual species should sum to total concentrations, total THM and total HAA heat maps would be expected to show similar characteristics to the individual species. Ex/em 350 nm/380 nm is observed in CNN heat maps for total THMs (Fig. 3a) and TCM (Fig. 3b). The secondary peak for total THMs at approximately ex/em 325 nm/450 nm is mirrored in the BDCM heat map (Fig. 3c). Similar conformance was not observed with MLP heat maps, for example, the areas of highest importance for BDCM (Fig. 4c) was not present in the total THM heat map (Fig. 4a). However, while some overlap is present between CNN heat maps of species and the total DBP levels, it should be noted that not all peaks are mirrored (e.g. BDCM peak at ex/em 250 nm/450 nm not seen in total THM map).

Individual species of DBPs showed differences between CNN heat maps. BDCM areas of importance were shifted to higher excitation and emission areas compared to TCM. A similar pattern can be seen between DCAA and TCAA. Identified differences in spectral areas between individual species were expected since preferential yields of specific by-products from pure model compounds have pointed to certain molecular structures resulting in the preferential formation of individual DBP species^38,39^. A shift to the greater importance of fluorophores at emissions > 450 nm could indicate BDCM and DCAA formation resulting from humic-like material with greater oxygen/carbon ratios and lower hydrogen/carbon ratios, implying an oxidation state ≥ 0^40^.

A second notable difference is the increased importance of protein-like material (ex/em 230–250 nm/300–350 nm) for HAA predictions. This peak location is typically associated with aromatic amino acids such as tryptophan and tyrosine^35^. Previous studies show that aromatic amino acids^41^ and protein-like fluorescence signals strongly correlate with HAA formation potentials^20,42,43^. In particular, the protein-like peak was observed to be most prominent for the prediction of TCAA. This observation conforms well to previous results that show higher TCAA formation than DCAA from aromatic amino acids that would contribute to the observed fluorescence signal^41^. From the MLP heat map of TCAA, regions surrounding the expected aromatic amino acid peak are positive. However, there is a strong negative relationship in the specific location of tryptophan fluorescence (ex/em 230 nm/340 nm).

## Discussion

This study investigated the use of deep CNNs to interpret fluorescence spectra and predict the formation of regulated chlorination DBPs from a drinking water treatment plant. The observed results indicate that deep CNNs are well suited to the task of interpreting fluorescence excitation–emission matrices and prediction of DBPs for several reasons: (1) overall prediction accuracy for all DBP groups and species were significantly reduced compared to MLP and previous modelling approaches using dimensionality reduction, (2) results from random initializations were less variable using deep CNNs compared to MLP and shallow CNNs, (3) deep CNN heat maps show trained networks utilize data from spectral regions that are well known to be associated with DBP formation potentials, and (4) compared to MLPs, CNNs show heat maps with characteristics more conformant with expectations of fluorescence from organic precursor material.

Compared to previous work that utilized dimensionality reduction prior to regression, the use of CNNs significantly improved the accuracy of prediction. Two commonly used dimensionality reduction methods, PARAFAC, PCA were applied for comparison as well as a factor regression using 3-way partial least squares (PLS) (Table 1). The components identified by these methods are discussed in more detail in a previous article using the same dataset^18^. In all cases, significant reduction of prediction accuracy was achieved using CNN architectures, particularly for HAA prediction. It is not straightforward to compare results to other studies given the variation in number of samples, methods for formation potential determination, range of concentrations in the training/test sets, and performance metrics. However, results found in this study also represent improvements over HAA and THM formation predictions previously reported using similar performance metrics (e.g. total THM MAE 13.5 µg/L and total HAA MAE 7.7 µg/L)^16^. Furthermore, the previous approaches to utilize fluorescence data for DBP predictions that have relied heavily on dimensionality reduction to identify relevant fluorescence features add complexity to the analysis process. In contrast, deep CNNs present an opportunity to utilize full fluorescence spectra without the need for manual or highly supervised feature selection through peak-picking, regional integrations, or PARAFAC analysis. It is thought that deep CNNs provide an opportunity for complex behaviours to be represented by several simpler representations. Observation of feature maps produced by convolutional layers show a hierarchy of feature representations, with general and smooth representations in high layers and progressively coarser and more specific highlighted spectral areas as increasing numbers of convolution and pooling layers are applied.

Neural networks are often discussed as black-box type algorithms, where the internal reasoning is unknown or difficult to illustrate. However, it is imperative that the logic of prediction algorithms used in applied tasks, such as prediction of potentially toxic disinfection by-products, is discernible. There is also an opportunity to use these powerful data-driven approaches to help identify important variables or characteristics of the system. The use of heat maps generated from an occlusion approach to identifying spectral areas that highly influence predictions gives insight into the decision-making process and helps confirm that trained networks are relying on data from spectral regions associated with DBP precursors. Furthermore, heat maps can help direct future more detailed studies investigating the characteristics of precursor material.

As such, the use of fluorescence spectroscopy coupled with machine learning techniques, such as deep CNNs, show promise to be used for rapid estimation of DBP formation potentials. In the context of typical regulatory thresholds for water treatment (total THMs < 80 µg/L, total HAAs < 60 µg/L) the presented methodology produced error levels (MAE 3.39–5.53 µg/L) that would be appropriate for rapidly informing operations and management regarding conformance with regulatory thresholds. Knowledge of DBP formation potentials in near real-time can enable tighter treatment controls and management efforts to minimize the public's exposure to DBPs.

## Methods

### Water samples

Water samples were obtained from parallel pilot treatment trains that were fed Otonabee River water (Peterborough, Ontario, Canada). Samples were obtained throughout the treatment train for fluorescence analysis and for determining DBP formation potentials. Processes applied included coagulation, flocculation, sedimentation, ozonation, advanced oxidation (peroxide and ozone), and filtration through anthracite or activated carbon. Further information on the pilot-scale set-up and water samples can be found in Peleato et al.^44^.

### Fluorescence

A total of 140 fluorescence spectra were collected using an Agilent Cary Eclipse fluorescence spectrophotometer (Mississauga, Canada). Excitation and emission wavelength ranges were 225–380 nm (5 nm increments), and 250–600 nm (2 nm increments), respectively. This resulted in fluorescence spectra with dimensions of 32 by 176, or 5632 total excitation/emission pairs. The fluorescence spectra were blank subtracted using Milli-Q^®^ water. The spectrum for Milli-Q^®^ water was also used to apply Raman corrections at an excitation wavelength of 350 nm and bandwidth of 5 nm to allow fluorescence intensities to be reported in Raman Units (RU)^45^. Absorbance values collected over the excitation–emission range were used to correct for inner-filter effects. Rayleigh scattering lines were removed by setting all values above 2nd order Rayleigh or below 1st order Rayleigh to 0. The absorbance corrected spectra were then scaled between 0 and 1 for each excitation/emission pair for input into MLPs or CNNs.

### Dimensionality reduction

PCA and PARAFAC were applied to identify a lower dimensional representation of fluorescence spectra. The two methods and resulting components are described further in Peleato et al.^18^. Briefly, PCA was carried out using R (V 3.2.5) on fluorescence spectra that were vectorized, mean centered and scaled to unit variance with respect to excitation/emission pairs. PARAFAC was applied using the drEEM toolbox for MATLAB and following the methodology described by Murphy et al.^24^. The PARAFAC model was validated using split-half validation where model consistency was checked between randomized dataset halves. The number of components for a valid PARAFAC model was found to be 5 and this number of components was also used to decide on the number of principal components used in regression. The loading plots of both PCA and PARAFAC are described and visualized in Peleato et al.^18^. The scores of each of the 5 components from PCA and PARAFAC were used as inputs into a MLP with 1 hidden layer and 100 nodes for regression.

3-way PLS was carried out using the PLS toolbox for MATLAB (Eigenvector Research). The normalized spectra used for both PARAFAC and PCA were utilized and the number of components was set to 5 for consistency with the dimensionality reduction models.

### Neural networks

All NNs were trained in Python 3.6 using the Keras library (v2.3.1; TensorFlow v1.15.0 backend). Hardware used was a Intel^®^ Xeon^®^ E2286G CPU and a NVIDIA GeForce RTX 2080. Training of each iteration of all models took less than 20 min.

Two general types of NNs were investigated: MLPs where there is a weighted connection between every node in subsequent layers, and CNNs. The number of nodes in each hidden layer of a MLP was defined as half of the previous layer. For example, with two hidden layers, hidden layer 1 would have 2816 nodes and layer 2 would have 1408.

**Figure 6:**
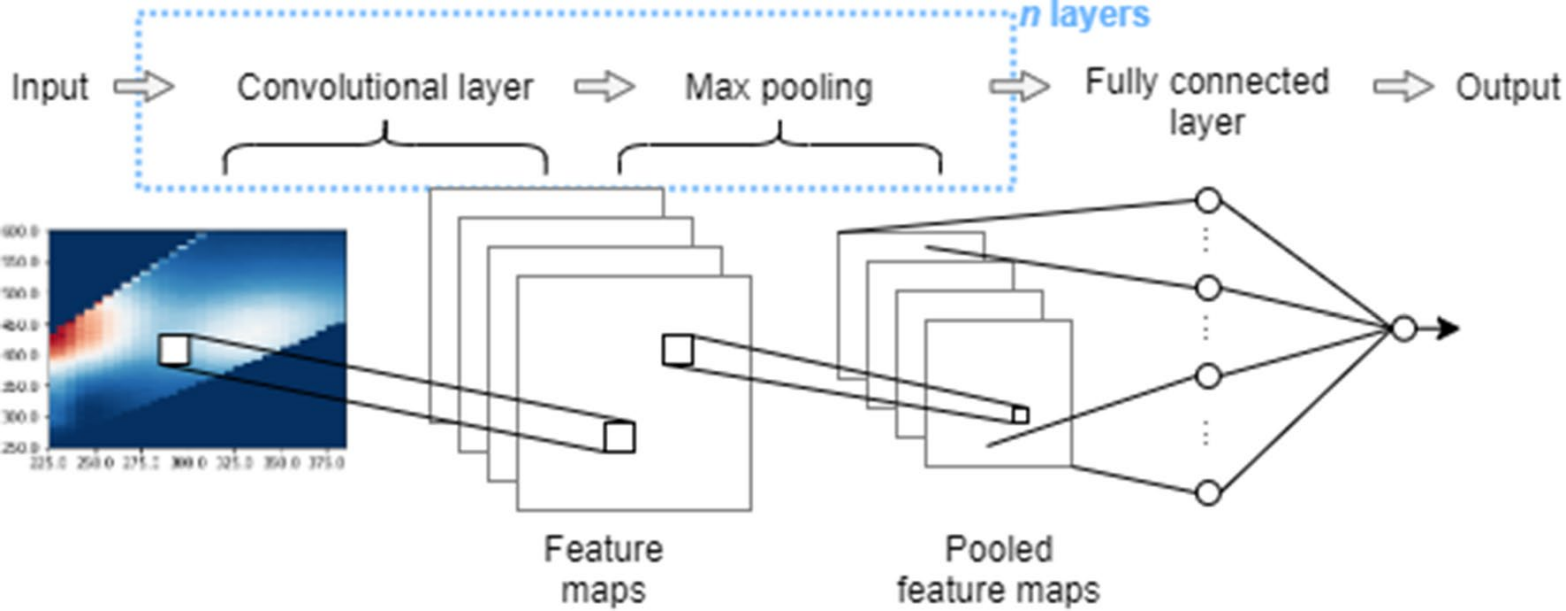
General schematic of convolutional neural network structure. The number of convolutional layers as well as the number of layers (convolution + max pooling) can be varied.

A general schematic of the CNN structure is shown in Fig. 6. Convolutional layers involved training a set of 2D filters or kernels, which are weighting functions multiplied with input values in a specific spatial window. 2D kernels were chosen to capture the 2D structure of the fluorescence excitation–emission spectra. Filters are smaller than the input dimensions and are slid across the entire input to produce feature mapping of the input. Since one filter or weighting function is used for the whole input space, fewer trainable parameters are needed than MLPs. It also gives rise to feature invariance since the trained filter can identify a feature in any position of the input space. Max pooling layers look for the maximum value within a spatial window and then uses that maximum value to represent the output over that spatial window, effectively reducing dimensionality. For more details on the mathematics of CNNs and the training process, see LeCun et al.^46^ and Goodfellow et al.^29^. CNN layers were considered as a set of convolutional layers followed by max pooling. Max pooling layers reduce the dimensionality of the features and provide an effective way to create hierarchies of detailed to general features. Several convolutional layers were set in series for some models, as this has been shown to provide an effective receptive field, which may provide advantages in chemometric applications^32^, while minimizing the number of trainable parameters^34^. Following convolutional layers, the structure was flattened, where pooled feature maps are vectorized into a dense hidden layer followed by a single output node. A varied number of structures were investigated to identify changes in performance based on depth (number of layers), size of convolutional filters (spatial window), number of max pooling layers, and number of convolutional filters. A summary of these structures is presented in Table 2.

**Table 2:** Descriptions of the general model types used. *n* refers to the number of layers and is varied based on model depth.

Model description	Structure
Multilayer perceptron	Input; dimensions = $(\# samples, \;5632)$
	*n* sets of:
	1. Dense ($nodes = 0.5 \cdot previous\;layer$)
	2. Batch normalization
	3. Activation (*elu*)
	Dense ($nodes = 1$)
CNN, no max pooling	Input; dimensions = $(\# samples, \;32{,} 176{,}1)$
	*n* sets of:
	1. Convolution 2D ($number \;of\; filters, \;size \;of\; filter \;(x,\; x)$)
	2. Batch normalization
	3. Activation (*elu*)
	Flatten
	Dense ($nodes = 1$)
CNN, with max pooling	Input; dimensions = $(\# samples, \;32{,} 176{,}1)$
	*n* sets of:
	1. Convolution 2D ($number \;of\; filters, \;size \;of\; filter\; (x, \;x)$)
	2. Batch normalization
	3. Activation (*elu*)
	4. Max pooling 2D ($2,2$)
	Flatten
	Dense ($nodes = 1$)
CNN *t* convolutions, 4 layers	Input; dimensions = $(\# samples, \;32{,} 176{,}1)$
	*t* sets of:
	*4* sets of:
	1. Convolution 2D ($number \;of\; filters, \;size \;of\; filter\; (x, \;x))$
	2. Batch normalization
	3. Activation (*elu*)
	Max pooling 2D (2, 2)
	Flatten
	Dense ($nodes = 1$)

Common between all structures and types of NNs was the use of batch normalization to speed up training^47^, followed by activation using an exponential linear unit (ELU) activation function (Eq. 1). The ELU function was chosen based on reports of greater learning rate and generalization for deep networks^48^. ELUs also avoiding issues with ‘dead’ nodes common with activations such as rectified linear units (ReLU) that have zero gradients below inputs of 0.

1 $$ELU:\;\;f(x) = \left\{ {\begin{array}{*{20}l} {x, } & {\quad x > 0} \\ {e^{x} - 1,} & {\quad x \le 0} \\ \end{array} } \right.$$

All networks were trained with a mean squared error loss function coupled with $L2$ regularization to prevent overfitting (Eq. 2). The Adam optimization algorithm was used for all training.

2 $$Loss = \frac{1}{N}\mathop \sum \limits_{i = 1}^{N} \left( {y_{i} - \widehat{{y_{i} }}} \right)^{2} + \lambda \left\| {w^{2} } \right\|$$

where, $y_{i}$ is the network output for sample $i$ (prediction), $\widehat{{y_{i} }}$ is the true value for sample *i*, *N* is the total number of samples, $\lambda$ is a hyperparameter to control $L2$ regularization (set to 0.01), $w$ are all the network weights.

Prediction accuracy was determined on a test set (20% of all data, n = 28) that was not used for training the network. The metric used to assess predictive performance was mean absolute error (MAE), primarily since it provides a metric in the same units used in analysis and is more easily interpreted. Statistical significance was determined using paired *t* tests comparing mean and standard deviation of MAEs with a confidence level of 95%.

### Occlusion method

An occlusion approach was used to generate heat maps of spectral areas that most influence prediction accuracy. After training a network, test data was modified by iteratively setting a spectral area or patch equal to 0. These occluded or corrupted training samples are then fed through the network to produce a prediction of DBP concentration (Table 3).

**Table 3:** Description of the occlusion method used to identify spectral heat maps or areas of importance.

Occlusion method	
1:	Train a network using original training data (*X*_*train*_)
2:	Predict outputs (*y*_*test*_) using original test data (*X*_*test*_)
3:	**For** *t* = *1* to *20,000* **do**
4:	Randomly select EEM patch from *X*_*test*_
5:	Set patch = 0 to create corrupted test set, *X*_*occluded*_
6:	Predict outputs (*y*_*occluded*_) using *X*_*occluded*_
7:	Average error calculated over all test data. $Error = \frac{1}{{N_{test} }}\sum\nolimits_{i = 1}^{{N_{test} }} {(y_{test} - y_{occluded} )}$, where *N*_*test*_ is the number of samples in the test set
8:	Check all excitation/emission were included in random selection
9:	Calculate average error over all iterations for each excitation/emission pair

The difference between initial predictions and occluded predictions provided an estimate of the importance of the occluded patch. If initial predictions and occluded predictions are identical or close, the trained network is not relying on that spectral area to estimate DBPs. On the other hand, if the error is high, the occluded region is influential on the accurate prediction of DBP levels.
